# Structural basis for the inhibition of coronaviral main proteases by PF-00835231: Crystal structures of M ^pro^ and M ^pro^ mutants bound to PF-00835231

**DOI:** 10.3724/abbs.2024122

**Published:** 2024-07-29

**Authors:** Xuelan Zhou, Xiaolu Lu, Cheng Lin, Xiaofang Zou, Wenwen Li, Xiangyi Zeng, Jie Wang, Pei Zeng, Weiwei Wang, Jin Zhang, Haihai Jiang, Jian Li

**Affiliations:** 1 College of Pharmacy Gannan Medical University Ganzhou 341000 China; 2 School of Basic Medical Sciences Jiangxi Medical College Nanchang University Nanchang 330031 China; 3 Shenzhen Crystalo Biopharmaceutical Co. Ltd. Shenzhen 518118 China; 4 Jiangxi Jmerry Biopharmaceutical Co. Ltd. Ganzhou 341000 China; 5 Shanghai Advanced Research Institute Chinese Academy of Sciences Shanghai 201204 China

**Keywords:** coronavirus, main protease, PF-00835231, crystal structure, inhibition

## Abstract

The main protease (M ^pro^) of coronaviruses plays a key role in viral replication, thus serving as a hot target for drug design. PF-00835231 is a promising inhibitor of SARS-CoV-2 M ^pro^. Here, we report the inhibitory potency of PF-00835231 against SARS-CoV-2 M ^pro^ and seven M ^pro^ mutants (G15S, M49I, Y54C, K90R, P132H, S46F, and V186F) from SARS-CoV-2 variants. The results confirm that PF-00835231 has broad-spectrum inhibition against various coronaviral M ^pro^s. In addition, the crystal structures of SARS-CoV-2 M ^pro^, SARS-CoV M ^pro^, MERS-CoV M ^pro^, and seven SARS-CoV-2 M ^pro^ mutants (G15S, M49I, Y54C, K90R, P132H, S46F, and V186F) in complex with PF-00835231 are solved. A detailed analysis of these structures reveals key determinants essential for inhibition and elucidates the binding modes of different coronaviral M ^pro^s. Given the importance of the main protease for the treatment of coronaviral infection, structural insights into M ^pro^ inhibition by PF-00835231 can accelerate the design of novel antivirals with broad-spectrum efficacy against different human coronaviruses.

## Introduction

In late 2019, a novel coronavirus disease caused by severe acute respiratory syndrome coronavirus 2 (SARS-CoV-2) was identified in Wuhan, China [ 1– 3]. SARS-CoV-2 belongs to the β-Coronaviridae family, which is in the same family as Middle East respiratory syndrome coronavirus (MERS-CoV) and SARS-CoV. All three strains are highly pathogenic [ 4 – 6]. However, SARS-CoV-2 easily mutates, and the resulting variants have raised concerns about the characteristics of the virus, including transmissibility and antigenicity. The World Health Organization (WHO) has identified five variants as variants of concern (VOCs), namely, B.1.1.7 (Alpha, α), B.1.351 (Beta, β), P.1 (Gamma, γ), B.1.617.2 (Delta, δ), and B.1.529 (Omicron), and several variants as variants of interest (VOI), including C.37 (Lambda, λ) ( https://www.who.int/activities/tracking-SARS-CoV-2-variants). The spread of SARS-CoV-2 as well as its variants has lasted for more than four years and has caused more than 774 million cases of COVID-19 as of 11 February 2024, of which 7.03 million have died ( https://covid19.who.int/). Many health agencies are looking for treatment options, and many drugs used for treating SARS-CoV-2 infection are also in clinical development [ 7 – 13]. One such strategy is targeting the main protease (M ^pro^) to selectively inhibit coronaviral replication [ 14 – 16]. 

The main coronaviral protease is also known as 3C-like protease (3CLpro) [ 15, 16]. After successful infection of host cells, the viral genome encodes two large overlapping polyproteins, namely, pp1a and pp1ab. M ^pro^ is able to process polyproteins to produce several nonstructural proteins (NSPs) necessary for viral replication. Additionally, M ^pro^ is highly conserved among β coronaviruses [16]. The recognition site of coronaviral M ^pro^ depends on Gln at the P1 position, and no proteases in humans share a similar cleavage site [ 14, 15]. Therefore, M ^pro^ of coronaviruses plays an important role in viral replication, and the selected M ^pro^ inhibitors should have broad-spectrum properties. 

To date, various inhibitors targeting coronaviral M ^pro^ have been developed by using drug discovery strategies such as high-throughput screening, structure-based drug design, and drug re-purposing [ 9, 12, 13, 15]. Among these, PF-07321332 and PF-00835231 represent state-of-the-art inhibitors with therapeutic potential [ 9 , 12, 17]. PF-07321332 shares structural similarity with GC376 and has been approved for clinical application in combination with ritonavir [ 9 , 18– 20]. PF-00835231 is an inhibitor designed for the main protease of SARS-CoV that emerged in 2003, but its clinical trials were suspended due to the rapid disappearance of the SARS outbreak [17]. Based on the high M ^pro^ similarity (96%) between SARS-CoV-2 and SARS-CoV, PF-00835231 could be a promising drug candidate for the treatment of SARS-CoV-2 infection [17]. Previous data have shown that PF-00835231 has a good inhibitory effect on SARS-CoV-2, but its oral bio-availability is relatively poor [21]. In this regard, there are two ways to increase its bio-availability. One way is to convert PF-00835231 into its phosphate pro-drug form (PF-07304814) as an intravenous treatment option, and another way is to optimize the PF-00835231 structure, largely based on the molecular basis of PF-00835231 in inhibiting various coronaviral M ^pro^s. 

In this study, we used a structure-based approach to investigate the inhibitory efficacy and molecular basis of M ^pro^ inhibition by PF-00835231. We found that PF-00835231 broadly inhibits SARS-CoV-2 M ^pro^ and its mutants (G15S, M49I, Y54C, K90R, P132H, S46F and V186F). These mutations can be found in different SARS-CoV-2 variants: B.1.351 Beta (K90R), C.37 Lambda (G15S), Delta AY.4 (Y54C), BA.5 Omicron (M49I), B.1.1.529 Omicron (P132H), B.1.1.529 Omicron (V186F), and B.1.1.529 Omicron (S46F). The crystal structures of M ^pro^s from SARS-CoV-2, SARS-CoV-2 VOC/VOIs, SARS-CoV and MERS-CoV M ^pro^s bound to PF-00835231 were solved, revealing the structural similarities and differences of PF-00835231 in binding with different M ^pro^s. These results provide structural insights into the precise inhibitory mechanism of this inhibitor against different M ^pro^s and strongly suggest that PF-00835231 has potential for the development of broad-spectrum drug candidates. Furthermore, this study provides critical information for the optimization of PF-00835231 and the design of more effective anti-coronaviral inhibitors. 

## Materials and Methods

### Expression and purification of coronaviral M ^pro^s 

According to previous experimental methods [ 19, 22], the genes encoding SARS-CoV-2, SARS-CoV, and MERS-CoV M ^pro^s were inserted into the pET-28a vector, and then the recombinant plasmids were constructed. The plasmid carrying the SARS-CoV-2 M ^pro^ gene was used as a template for site-directed mutagenesis to generate a variety of SARS-CoV-2 M ^pro^ mutants, including G15S, S46F, M49I, Y54C, K90R, P132H, and V186F. Then, the recombinant plasmids were introduced into competent *E. coli* Rosetta DE3 cells for protein expression. The expression and purification of wild-type M ^pro^ and M ^pro^ mutants were carried out according to methods described in previous articles [ 19, 22]. Briefly, the recombinant plasmid was transformed into *E*. *coli* BL21 (DE3) cells and the transformed bacteria were cultivated in Luria Broth (LB), a final concentration of 0.5 mM isopropyl-d-1-thiogalactopyranoside (IPTG) was added to induce the expression of M ^pro^ proteins. Purification was performed using a HisTrap column (Cytiva, Tokyo, Japan) and the target protein was eluted by imidazole gradient treatment. Furthermore, the TEV protease was used to remove the N-terminal His tag. 

### Enzymatic inhibition assay

Fluorogenic substrates used as donor and quencher pairs were commercially synthesized. The inhibitory efficacies of PF-00835231 against different coronaviral M ^pro^ proteins were detected using fluorescence resonance energy transfer (FRET)-based enzymatic assays, which have been reported previously [23]. Briefly, PF-00835231 was dissolved in DMSO to prepare a stock solution (10 mM) in advance. Then, PF-00835231 was subjected to a 3-fold serial dilution in triplicate and incubated with different coronaviral M ^pro^ proteins and SARS-CoV-2 M ^pro^ mutants for 30 min at 25°C. Subsequently, a FRET substrate was added to the reaction system which contain protein, PF-00835231 and reaction buffer (50 mM Tris, 1 mM EDTA), followed by another 20 min of incubation. The system was monitored with a microplate reader (SpectraMax Paradigm; Molecular Devices, San Jose, USA), and the fluorescence was recorded during the reaction. The inhibition activities (%) of PF-00835231 against coronaviral M ^pro^s were finally determined using GraphPad Prism (GraphPad software, La Jolla, USA). 

### Crystallization

The recombinant M ^pro^ proteins were concentrated to 10 mg/mL and incubated on ice with PF-00835231 at a 1:5 molar ratio for 30 min. Crystallization was carried out at 18°C by using the hanging drop vapor-diffusion method [24]. After 3 to 5 days, crystals of M ^pro^s in complex with PF-00835231 were obtained. The final crystallization conditions for the SARS-CoV-2 M ^pro^-PF-00835231 complex were 0.1 M HEPES sodium (pH 7.5), 10% v/v 2-propanol, and 20% w/v PEG 4000. The final crystallization conditions for the SARS-CoV M ^pro^-PF-00835231 complex were 0.1 M HEPES (pH 7.5), 12% w/v PEG 8000, and 10% w/v ethylene glycol. The final crystallization conditions for the MERS-CoV-M ^pro^-PF-00835231 complex were 0.2 M sodium chloride and 20% w/v PEG 3350. The final crystallization conditions for the SARS-CoV-2 M ^pro^ (Y54C)-PF-00835231 complex were 0.7 M sodium citrate tribasic dihydrate and 0.1 M Bis-Tris propane, pH 7.0. The final crystallization conditions of other SARS-CoV-2 M ^pro^ mutants (including G15S, S46F, M49I, K90R, P132H, and V186F) in complex with PF-00835231 were 0.1 M-0.25 M Na _2_SO _4_ and 20% w/v to 25% w/v PEG3350. 

### Data collection, structure determination, and refinement

Before the data were collected, the crystals were soaked in a cryoprotective solution containing 20% glycerol with crystallization conditions and then stored in liquid nitrogen. Diffraction data were collected at 100 K on the macromolecular crystallographic beamlines 02U1 (BL02U1) and 10U2 (BL10U2) at the Shanghai Synchrotron Radiation Facility (SSRF, Shanghai, China). All the datasets were processed using HKL2000 software [25]. The phase problem was solved by molecular replacement. The structures were refined for several cycles in Phenix to achieve the desired resolution [26]. 

## Results

### Broad-spectrum inhibitory activity of PF-00835231

**Figure 1:**
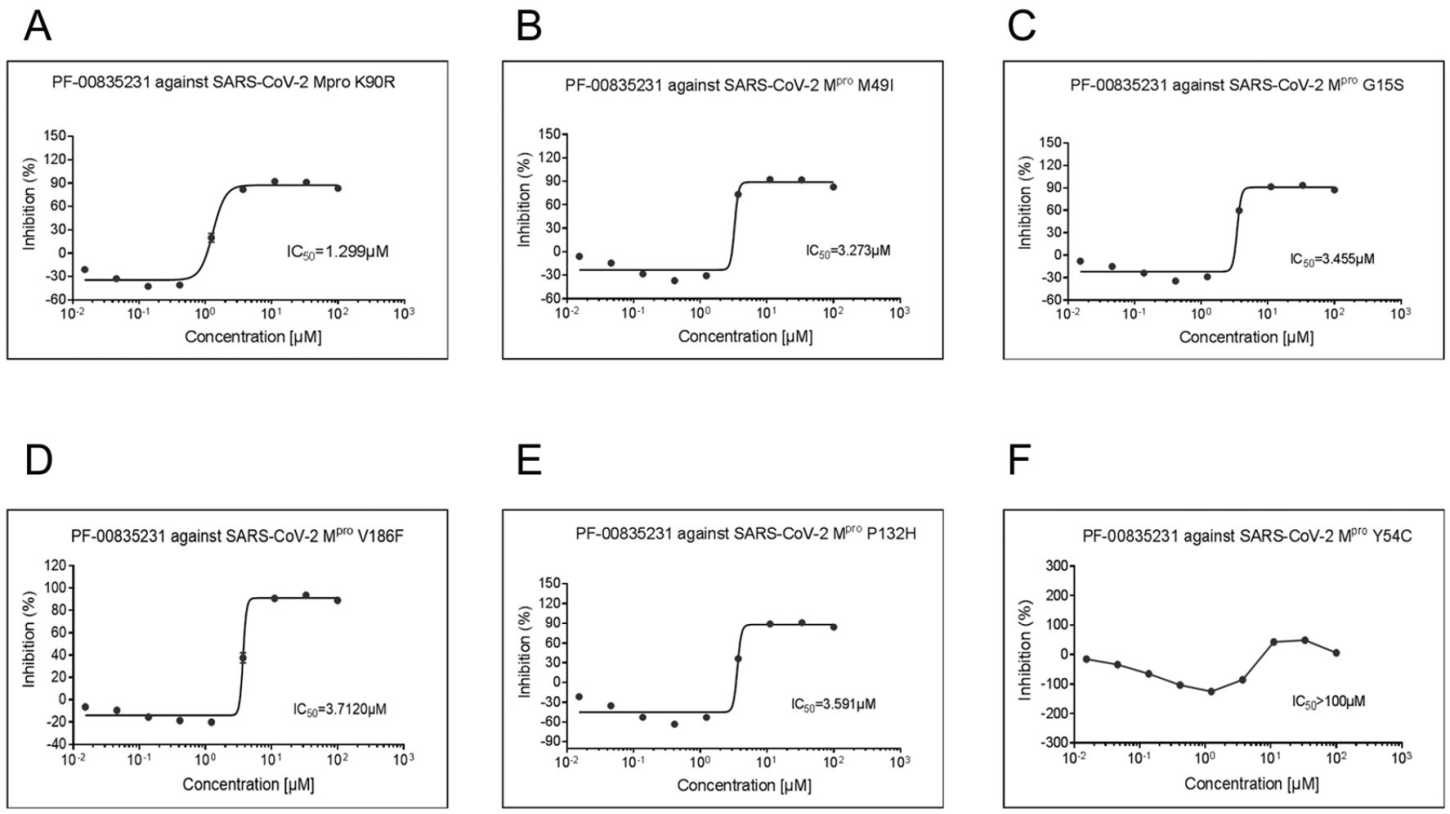
Figure 1 Enzymatic inhibition of SARS-CoV-2 M ^pro^ and SARS-CoV-2 M ^pro^ mutants by PF-00835231 (A) Inhibitory potency of PF-00835231 against the main protease of the SARS-CoV-2 Mpro K90R. (B) Inhibition of PF-00835231 by the main protease of SARS-CoV-2 Mpro M49I. (C) Inhibition of the main protease of SARS-CoV-2 Mpro G15S by PF-00835231. (D) Inhibition of PF-00835231 against the main protease of SARS-CoV-2 Mpro V186F. (E) Inhibition of PF-00835231 against the main protease of SARS-CoV-2 Mpro P132H. (F) Inhibition of PF-00835231 against the main protease of SARS-CoV-2 Mpro Y54C.

The SARS-CoV-2 M ^pro^ and M ^pro^ mutants (G15S, M49I, Y54C, K90R, P132H, S46F and V186F) variants were expressed and purified as previously reported [ 19 , 22]. The inhibitory activities of PF-00835231 against M ^pro^s were detected by using a fluorescence resonance energy transfer (FRET) assay. The results showed that PF-00835231 can effectively inhibit SARS-CoV-2 M ^pro^ and its mutants. The IC _50_ value of PF-00835231 against SARS-CoV-2 M ^pro^ was 0.0086 μM, while the IC _50_ value of PF-00835231 against SARS-CoV-2 M ^pro^ mutants, including K90R ( Figure 1A), M49I ( Figure 1B), G15S ( Figure 1C), V186F ( Figure 1D), P132H ( Figure 1E) and Y54C ( Figure 1F), ranged from 1.2 to 3.7 μM. Based on our previous work, PF-00835231 also inhibits M ^pro^s of HCoV-NL63, HCoV-HUK1, MERS-CoV, and SARS-CoV [27]. The Ki values of PF-00835231 against M ^pro^s from various coronaviruses, including HCoV-NL63, HCoV-229E, PEDV, FIPV, HKU4-CoV, HCoV-OC43, and HCoV-HKU1, range from 30 pM to 4 nM [12]. In addition, PF-00835231 exerts antiviral activity against SARS-CoV and HCoV-229E with very low semi-maximum effective concentration (EC _50_) values [17]. These data suggest that PF-00835231 has a broad enzymatic inhibitory effect against coronavirus M ^pro^s and that this compound has potential for preventing current and future coronavirus pandemics.

### Crystal structure of SARS-CoV-2 M ^pro^ in complex with PF-00835231 

**Figure 2:**
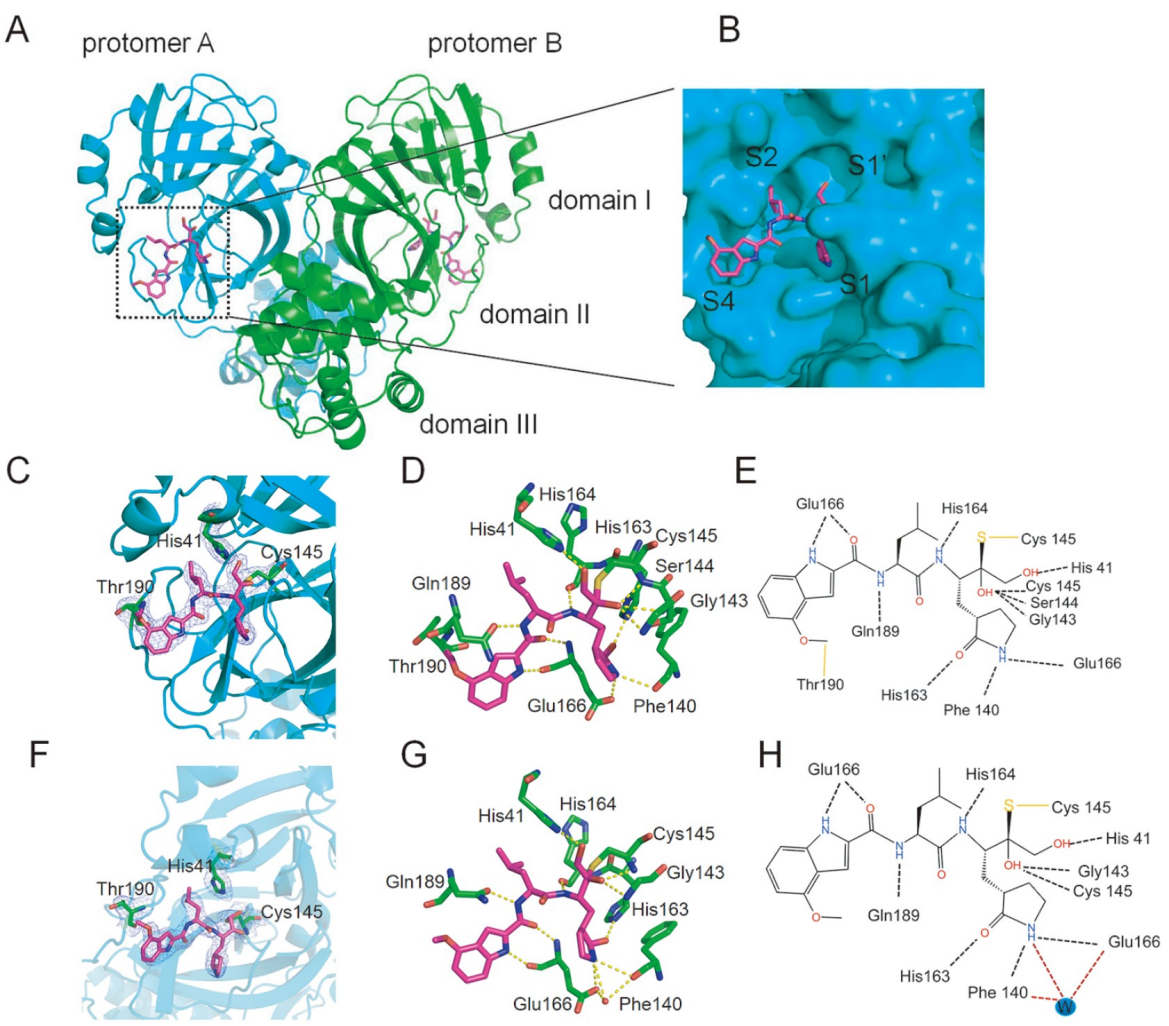
Figure 2 Crystal structure of SARS-CoV-2 M ^pro^ in complex with PF-00835231 (A) Overall structure of the SARS-CoV-2 Mpro-PF-00835231 complex. Mpro is shown as a cartoon. Three subdomains and two propolymers of the main protease are labeled. PF-00835231 is shown as sticks with carbon atoms in magenta, oxygen atoms in red, and nitrogen atoms in blue. (B) An enlarged view of the substrate binding pocket of SARS-CoV-2 Mpro with the protease shown as a surface and the inhibitor shown as a stick plot.(C–E) SARS-CoV Mpro-PF-00835231 complex on the A chain. (F–H) SARS-CoV Mpro-PF-00835231 complex on the B chain. (C,F) 2Fo-Fc electron density map contoured at 1.0σ. (D,E) Schematic interaction between PF-00835231 and SARS-CoV-2 Mpro. Hydrogen-bonding interactions are indicated by dashed lines, and one water molecule is labeled W. (G,H) The detailed interaction between PF-00835231 and SARS-CoV-2 Mpro with residues involved in inhibitor binding (within 3.5 Å) is highlighted. One water molecule is labeled W, and hydrogen bond interactions are despicted as dashed lines.

To understand the mechanism by which PF-00835231 inhibits SARS-CoV-2 M ^pro^, we solved the crystal structure of SARS-CoV-2 M ^pro^ in complex with PF-00835231. The resolution of the complex structure is 2.21 Å. Data collection and refinement statistics are summarized in Table 1. As shown in Figure 2A, SARS-CoV-2 M ^pro^ is a homodimer in the complex structure and can be divided into three subdomains, namely, domain I (residues 3 to 99), domain II (residues 100 to 199), and domain III (residues 201 to 300). Domains II and III are connected by a long ring (residues 175 to 200). The narrow cavity between domain I and domain II contains the inhibitor PF-00835231, which is present in both protomer A and protomer B. An enlarged view of the narrow cavity revealed that PF-00835231 occupied the S1, S1’, and S2 subsites of SARS-CoV-2 M ^pro^ in an extended conformation ( Figure 2 B).

**Table:** **Table 1** Data collection and refinement statistics

	MERS-PF-00835231	SARS-PF-00835231	SARS-CoV-2-PF-00835231	SARS-CoV-2-G15S-PF-00835231	SARS-CoV-2- M49I-PF-00835231
Data collection	8J34	8Z1H	8J32	8J35	8J36
Beam line	BL02U1	BL02U1	BL10U2	BL10U21	BL10U21
Wavelength (Å)	0.97918	0.97918	0.97918	0.97918	0.97918
Space group	P212121	P1	P1211	P1211	P1211
a, b, c (Å)	57.90,91.33,118.14	55.04,60.93,68.22	55.17,98.88,58.81	55.57,99.02,59.54	55.51,99.25,59.69
α, β, γ (°)	90., 90.,90.	91.23,102.46,108.66	90.,108.00,90.	90.,108.49,90.	90.,108.38,90.
Total reflections	264915	78284	122548	301714	416740
Unique reflections	28551	24344	28537	49430	65201
Resolution (Å)	2.30(2.43–2.30)	2.61(2.68–2.61)	2.21(2.33–2.21)	1.79(1.89–1.79)	1.65(1.74–1.65)
R-merge (%)	8.5(39.8)	7.2(13.6)	11.3(62.3)	2.6(68.6)	4.4(63.1)
Mean I/σ (I)	18.5/7.0	11.3/6.0	8.7/2.3	19.4/2.4	20.6/2.6
Completeness (%)	100.0(100.0)	98.2(97.2)	94.4(95.7)	86.0(82.0)	89.0(98.7)
Redundancy	9.3(9.8)	3.2(2.6)	4.3(4.3)	6.1(6.1)	6.4(5.5)
Refinement					
Resolution (Å)	51.99–2.30	32.96–2.61	46.01–2.21	52.70–1.79	46.59–1.65
Rwork/Rfree (%)	19.82/25.55	18.89/23.63	20.44/25.42	20.77/24.66	21.19/23.44
Atoms	4675	4490	4564	4555	4616
Mean temperature factor (Å2)	35.7	32.9	40.9	32.8	29.5
Rmsd bond lengths (Å)	0.008	0.008	0.007	0.007	0.006
Rmsd bond angles (°)	1.118	1.048	0.973	0.876	0.904
Ramachandran plot (%)					
Preferred	97.81	97.73	97.13	98.14	97.63
Allowed	2.19	2.27	2.87	1.86	2.37
outliers	0	0	0	0	0
Rpim	0.029/0.133	0.048/0.109	0.060–0.326	0.023/0.297	0.019/0.298
CC1/2	0.998/0.976	0.975/0.425	0.994/0.835	0.999/0.826	0.999/0.774
Search model	7DR8	7DQZ	7C2Q	7C2Q	7C2Q
RSCC	0.89	0.91	0.84	0.93	0.89
0.91	0.95	0.86	0.94	0.93

*^a^* The values in parentheses are for the outermost shell.

*^b^* R _free_ is the R _work_ based on 5% of the data excluded from the refinement.

R _work_= *h*klF _obs_ –|F _calc_||/ *h*kl|F _obs_|; where F _obs_ and F _calc_ are the observed and calculated structure factors, respectively.

A difference was found in the binding patterns of the inhibitor with the two protomers of the dimer, and the difference mainly existed in the indole group ( Figure 2C–H). According to the electron density maps of both the A ( Figure 2C) and B protomers ( Figure 2F), PF-00835231 forms an additional C-S covalent bond with the sulfur atom of Cys145. In addition, the carbon on the methoxyl group of the indole group was found to interact with Thr190. To further reveal the mechanism of SARS-CoV-2 M ^pro^ inhibition by PF-00835231, we analyzed the interactions between PF-00835231 and SARS-CoV-2 M ^pro^ in detail ( Figure 2). PF-00835231 employs a hydroxymethyl ketone component as a directional warhead. The 4-hydroxy group of PF-00835231 occupies the S1’ pocket and forms a hydrogen bond with the main chain of His41 in M ^pro^. In addition, residues Gly143, His163 and Cys145 of SARS-CoV-2 M ^pro^ form hydrogen bonds with the hydroxyl groups of PF-00835231. The lactam ring of PF-00835231 occupies the S1 pocket of M ^pro,^ and the nitrogen of the lactam ring forms hydrogen bonds with Phe140 and Glu166 directly or via a water molecule (W) when the carbyl oxygen of lactam forms a hydrogen bond with the nitrogen of His163. While the leucine moiety of PF-00835231 occupies the S2 pocket, the indole group of PF-00835231 occupies the S4 pocket of M ^pro^. The nitrogen of the indole group and the carbyl oxygen of the main chain form a hydrogen bond with Glu166, while the nitrogen of the main chain forms a hydrogen bond with His164. These differences can be seen on both the A ( Figure 2C,E) and B ( Figure 2F,H) chains, and they differ in the indole group. The carbon of the indole methoxy group is covalently bound to Thr190, which is specific to the A-chain. We found that the indole-linked methoxyl group has some interactions with Thr190, and we speculated that the oxymethyl carbon may form a covalent interaction with the carbon of Thr190. Because the electron density is not sufficient, the specific effect needs to be further verified, but it is certain that there is a certain interaction. The covalent binding of 5231 to Thr190 occurs in chain A but not in chain B, possibly because the location is dynamic. Therefore, covalent binding to Thr190 can be observed, suggesting a novel mechanism of SARS-CoV-2 M ^pro^ inhibition by PF-00835231. 

Previous reports also solved the crystal structure of SARS-CoV-2 M ^pro^ in complex with PF-00835231 (PDB ID 8DSU and 6XHM) [ 17 , 28]. By superimposing the structure of the SARS-CoV-2 M ^pro^-PF-00835231 reported in this study ( Supplementary Figure S1A) complex with the previously solved structures, the RMSD on the 433 optimally arranged Cα atoms are 0.653 Å (PDB ID 6XHM; Supplementary Figure S1B) and 0.53 Å (PDB ID 8DSU; Supplementary Figure S1C), respectively. Through The zoomed-in view of the substrate binding pocket of main proteases ( Supplementary Figure S1D–F) and the 2Fo-Fc electron density map (contoured at 1.0σ) of the inhibitor ( Supplementary Figure S1G–I), the binding pattern of PF-00835231 with SARS-CoV-2 M ^pro^ is highly similar, except the binding mode of the indole group. Covalent binding was not detected between the indole group of PF-00835231 and Thr190 of the main protease in previous studies [ 17, 28]. 

### Crystal structure of SARS-CoV-2 M ^pro^ mutants in complex with PF-00835231 

**Figure 3:**
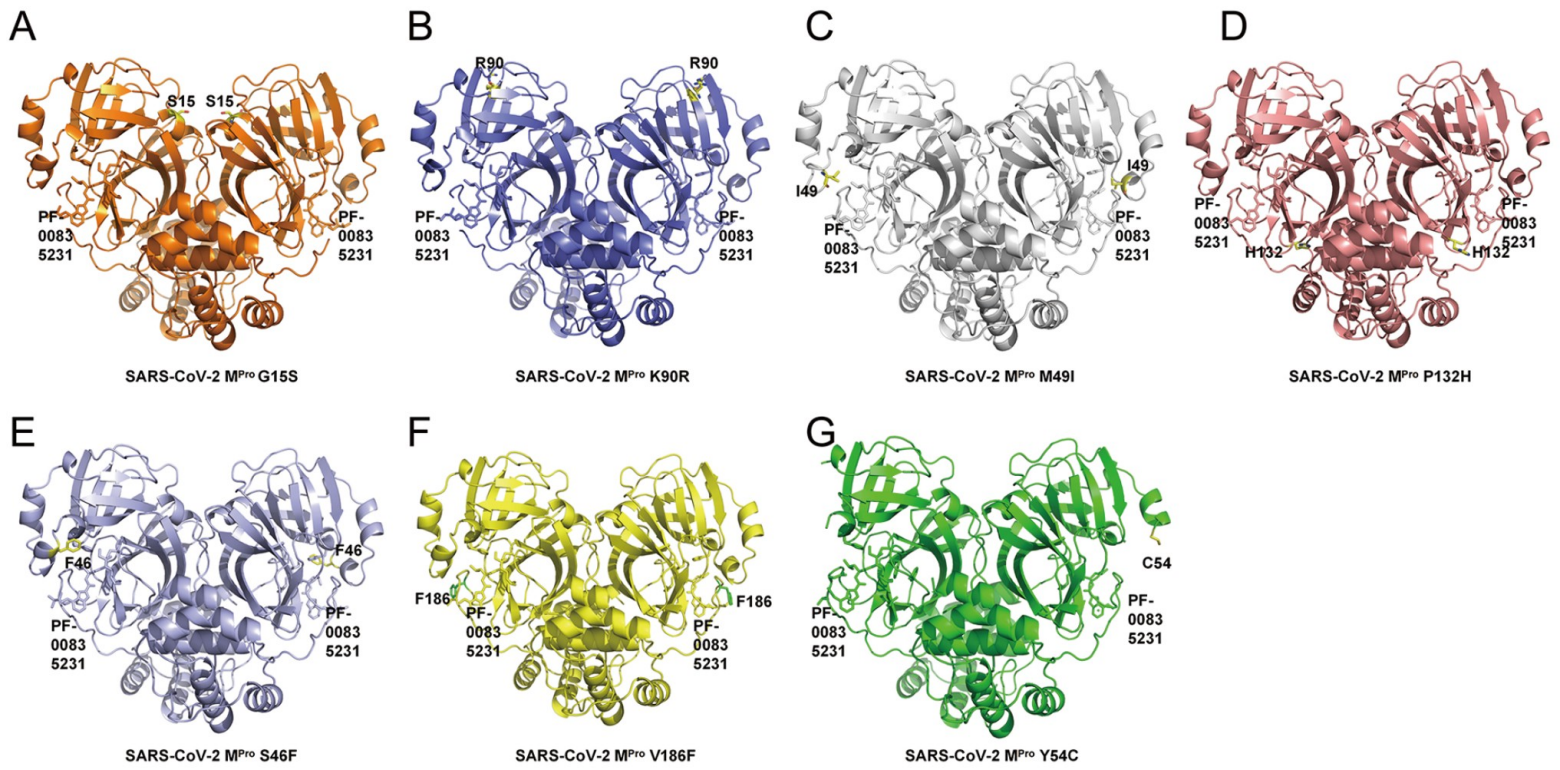
Figure 3 Structural overview of M ^pro^ mutants from SASR-CoV-2 in complex with PF-00835231 (A) Overall structure of the Mpro G15S-PF-00835231 complex. The G15S mutant is shown as an orange cartoon, while PF-00835231 is displayed as a stick. (B) Overall structure of the Mpro K90R-PF-00835231 complex. K90R mutant in slate cartoons, while PF-00835231 is displayed in stick representation. (C) Overall structure of the Mpro M49I-PF-00835231 complex. The M49I mutant is shown as a gray 90 cartoon, while PF-00835231 is displayed as a stick. (D) Overall structure of the Mpro P132H-PF-00835231 complex. P132H mutant in a salmon cartoon, while PF-00835231 is displayed as a stick. (E) Overall structure of the M pro S46F-PF-00835231 complex. The S46F mutant is shown as a light blue cartoon, while PF-00835231 is displayed as a stick. (F) Overall structure of the Mpro V186F-PF-00835231 complex. The V186F mutant is shown as a yellow cartoon, while PF-00835231 is displayed as a stick. (G) Overall structure of the Mpro Y54C-PF-00835231 complex. The Y54C mutant is shown as a green cartoon, while PF-00835231 is displayed as a stick.

We then used the co-crystallization method to determine the crystal structures of several M ^pro^ mutants of SARS-CoV-2 in complex with PF-00835231 ( Figure 3). The resolutions for these structures are 1.79 Å (M ^pro^ G15S with PF-00835231; Figure 3)A, 1.68 Å (M ^pro^ K90R with PF-00835231; Figure 3B), 1.65 Å (M ^pro^ M49I with PF-00835231; Figure 3C), 1.72 Å (M ^pro^ P132H with PF-00835231; Figure 3D), 1.64 Å (M ^pro^ S46F with PF-00835231; Figure 3E), 1.66 Å (M ^pro^ V186F with PF-00835231; Figure 3F) and 1.91 Å (M ^pro^ Y54C with PF-00835231; Figure 3G), respectively. The data collection and detailed statistics are shown in Table 1 and Table 2. In each of these seven complex structures, each SARS-CoV-2 M ^pro^ mutant molecule appears as a dimer form, which is also the form with enzymatic activity. Overall, each protomer of the SARS-CoV-2 M ^pro^ mutant binds to one PF-00835231 molecule.

**Table:** **Table 2** Data collection and refinement statistics

	SARS-CoV-2- K90R-PF-00835231	SARS-CoV-2- P132H-PF-00835231	SARS-CoV-2- S46F-PF-00835231	SARS-CoV-2- V186F-PF-00835231	SARS-CoV-2- Y54C-PF-00835231
Data collection	8J37	8J38	8J3B	8J39	8J3A
Beam line	BL10U2	BL10U2	BL10U2	BL10U2	BL10U2
Wavelength (Å)	0.97918	0.97918	0.97918	0.97918	0.97918
Space group	P1211	P1211	P1211	P1211	P1211
a, b, c (Å)	55.63,98.98,59.56	55.46,99.30,59.25	55.46,99.30,59.25	55.45,99.05,59.26	54.87,99.93,58.71
α, β, γ (°)	90,108.71,90	90,108.41,90	90,108.41,90	90,108.37,90	90,106.94,90
Total reflections	411159	372487	342391	400517	285620
Unique reflections	65079	57473	70413	71629	46688
Resolution (Å)	1.68(1.77–1.68)	1.72(1.81–1.72)	1.64(1.72–1.64)	1.66(1.75–1.66)	1.91(2.02–1.91)
R-merge (%)	5.9(64.5)	5.1(61.4)	4.4(23.4)	6.4(59.7)	6.2(48.8)
Mean I/σ (I)	16.5/2.5	21.1/2.8	19.0/2.8	14.7/2.6	14.4/3.0
Completeness (%)	93.8(100.0)	88.5(99.8)	94.2(98.8)	99.5(100.0)	99.8(100.0)
Redundancy	6.5(5.7)	6.5(5.9)	4.9(2.8)	5.6(4.9)	6.1(5.1)
Refinement					
Resolution (Å)	46.51–1.68	56.21–1.72	48.98–1.64	46.41–1.66	37.33–1.91
Rwork/Rfree (%)	21.36/24.17	21.08/24.05	21.10/23.61	21.45/25.10	20.04/23.54
Atoms	4640	4623	4949	4700	4441
Mean temperature factor (Å ^2^)	27.8	27.7	27.6	27.5	40.3
Rmsd bond lengths (Å)	0.006	0.007	0.007	0.006	0.007
Rmsd bond angles (°)	0.887	0.891	0.853	0.894	0.902
Ramachandran plot (%)					
Preferred	97.47	97.47	97.98	97.64	97.88
Allowed	2.53	2.53	2.02	2.36	2.12
Outliers	0	0	0	0	0
Rpim	0.025/0.298	0.022/0.276	0.021/0.172	0.030/0.301	0.027/0.242
CC1/2	0.999/0.835	0.999/0.823	0.998/0.911	0.998/0.794	0.021/0.172
Search model	7C2Q	7C2Q	7C2Q	7C2Q	7C2Q
RSCC	0.90	0.89	0.92	0.90	0.90
0.92	0.92	0.93	0.92	0.93

*^a^* The values in parentheses are for the outermost shell.

*^b^* R _free_ is the R _work_ based on 5% of the data excluded from the refinement.

R _work_= *h*klF _obs_ –|F _calc_||/ *h*kl|F _obs_|; where F _obs_ and F _calc_ are the observed and calculated structure factors, respectively.

**Figure 4:**
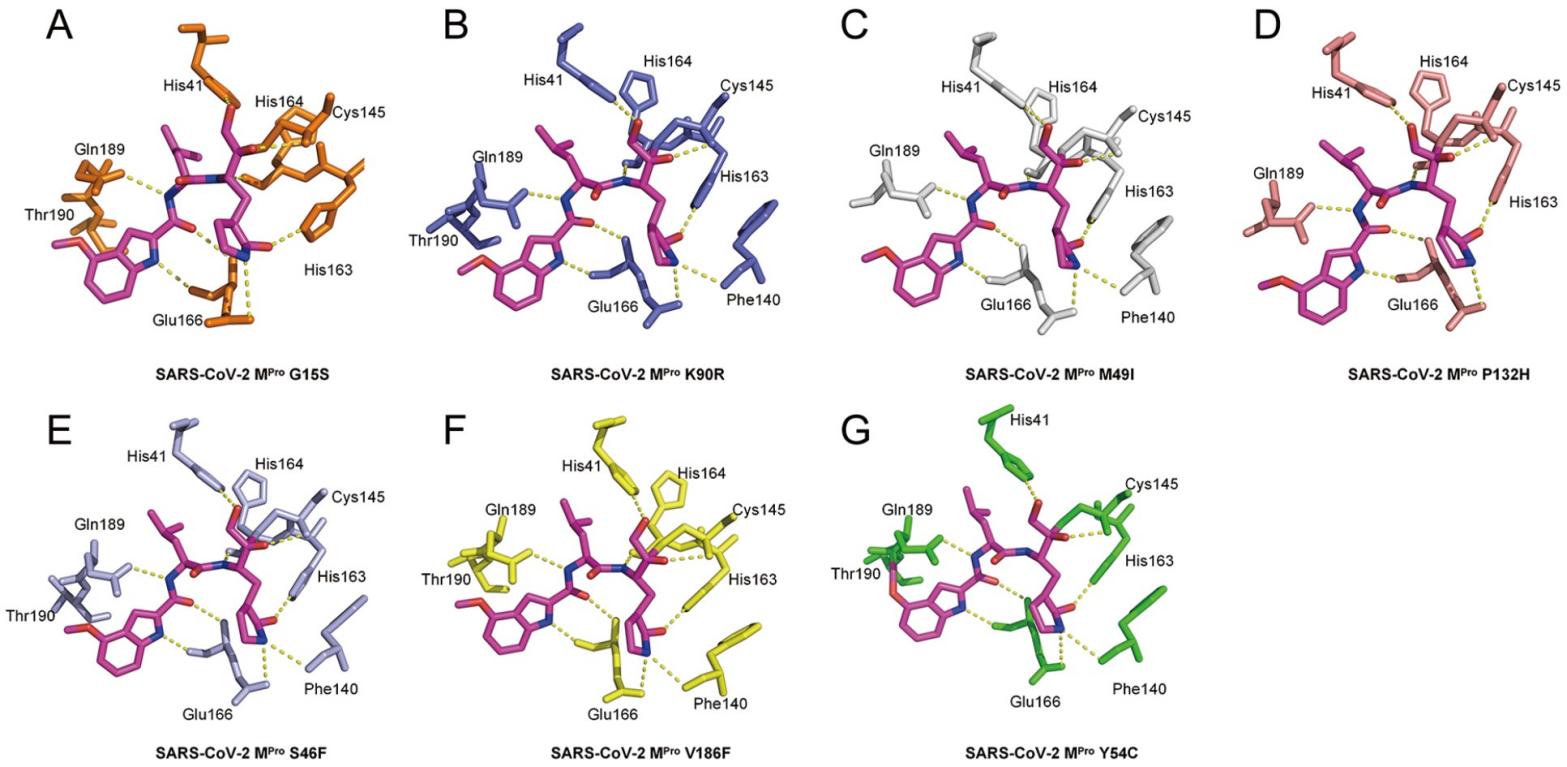
Figure 4 Interaction details between different M ^pro^ mutants and PF-00835231 PF-00835231 is shown as sticks with carbon atoms in magenta, oxygen atoms in red, and nitrogen atoms in blue. (A) Interaction between Mpro G15S and the PF-00835231 complex. (B) Interaction between Mpro K90R and PF-00835231. (C) Interaction between Mpro M49I and PF-00835231. (D) Interaction between Mpro P132H and PF-00835231. (E) Interaction between Mpro S46F and PF-00835231. (F) Interaction between Mpro V186F and PF-00835231. (G) Interaction between Mpro Y54C and PF-00835231.

To further investigate the details of the interaction between PF-00835231 and different mutants, the amino acids that interact with PF-00835231 are clearly labeled as stick patterns ( Figure 4). The interaction details between M ^pro^ G15S and PF-00835231 show that PF-00835231 interacts with several residues, including His41, Cys145, His163, His164, Glu166, and Gln189 ( Figure 4A). The interaction details between M ^pro^ K90R and PF-00835231 show that PF-00835231 interacts with several residues, including His41, Phe140, Cys145, His163, His164, Glu166, and Gln189 ( Figure 4B). The details of the interaction between M ^pro^ M49I and PF-00835231 show that PF-00835231 interacts with several residues, including His41, Phe140, Cys145, His163, His164, Glu166, and Gln189 ( Figure 4C). The details of the interaction between M ^pro^ P132H and PF-00835231 show that PF-00835231 interacts with several residues, including His41, Phe140, Cys145, His163, His164, Glu166, and Gln189 ( Figure 4D). The details of the interaction between M ^pro^ S46F and PF-00835231 show that PF-00835231 interacts with several residues, including His41, Phe140, Cys145, His163, His164, Glu166, and Gln189 ( Figure 4E). The details of the interaction between M ^pro^ V186F and PF-00835231 show that PF-00835231 interacts with several residues, including His41, Phe140, Cys145, His163, His164, Glu166, and Gln189 ( Figure 4F). The interaction details between M ^pro^ Y54C and PF-00835231 show that PF-00835231 interacts with several residues, including His41, Phe140, Cys145, His163, His164, Glu166 and Gln189 ( Figure 4G). Interestingly, in the M ^pro^ Y54C-PF-00835231 complex, the inhibitor covalently binds to Gln189, not Thr190, which is different from what is observed in the SARS-CoV-2 M ^pro^-PF-00835231 complex. This further confirmed the ability of PF-00835231 to form covalent bonds with the residue in the S4 pocket of the main protease.

**Figure 5:**
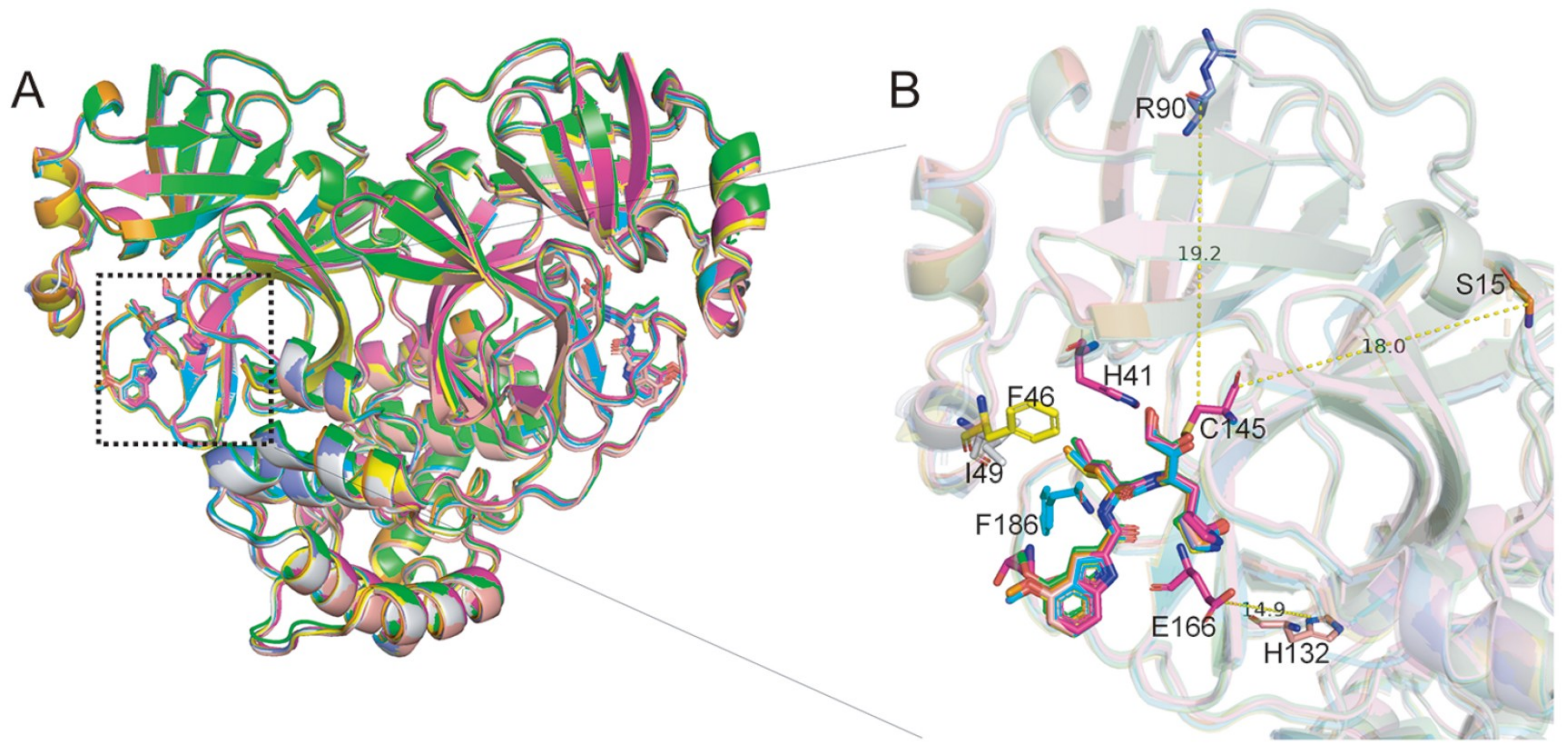
Figure 5 Structural comparison of PF-00835231 bound to SARS-CoV-2 M ^pro^ mutants (A) Overall structure of the SARS-CoV-2 Mpro mutant-PF-00835231 complex. SARS-CoV-2 Mpro-PF-00835231 is shown as a cartoon in light magenta, and Y54C (green), V168F (yellow), S46F (light blue), P132H (salmon), M49I (gray), K90R (slate), and G15S (orange) are shown. (B) The zoomed-in view of structural superpositions with the location and distance of the mutant residues relative to the binding site highlighted. PF-00835231 and the mutant residues are shown as sticks.

To compare the conformational changes of the M ^pro^ mutant-inhibitor complexes with those of the wild-type M ^pro^-inhibitor complex, we superimposed these structures ( Figure 5, Supplementary Figure S2 and Supplementary Figure S3). The results clearly showed that the ligand binding patterns are not disrupted by these mutations ( Figure 5A). The key hydrogen bond interactions between residues in wild-type M ^pro^ and PF-00835231 are consistent with the structure of wild-type M ^pro^-PF-00835231, except Phe140, Gln189 and Thr190. PF-00835231 was not found to interact with Phe140 in M ^pro^ G15S or M ^pro^ P132H and was not found to interact with Thr190 in M ^pro^ mutants. PF-00835231 was found to covalently interact with Gln189 in M ^pro^ Y54C, which is probably due to its dynamic nature. In fact, the G15S, K90R, and P132H mutations are located far from the PF-00835231 binding pocket ( Figure 5B). For further comparison, we analyzed that the overall structural comparison between SARS-CoV-2 M ^pro^-PF-00835231 and different mutants-PF-00835231 in detail (G15S ( Supplementary Figure S2A), K90R ( Supplementary Figure S2B), M49I ( Supplementary Figure S2C), P132H ( Supplementary Figure S3A, S46F ( Supplementary Figure S3B), V186F ( Supplementary Figure S3C) and Y54C ( Supplementary Figure S3D). We analyzed not only the zoomed-in view of the substrate binding pocket of main proteases [G15S ( Supplementary Figure S2D), K90R ( Supplementary Figure S2E) , M49I ( Supplementary Figure S2F), P132H ( Supplementary Figure S2E), S46F ( Supplementary Figure S3F),V186F ( Supplementary Figure S3G) and Y54C ( Supplementary Figure S3H)] but also the 2Fo-Fc electron density maps of the PF-00835231 bound to different SARS-CoV-2 M ^pro^ mutants [G15S ( Supplementary Figure S2G), K90R ( Supplementary Figure S2H) , M49I ( Supplementary Figure S2I), P132H ( Supplementary Figure S3I), S46F ( Supplementary Figure S3J),V186F ( Supplementary Figure S3K) and Y54C ( Supplementary Figure S3L)]. The crystal structure revealed in this study also showed that the mutation does not cause any significant change in the inhibitory potency of this inhibitor.

### Crystal structures of SARS-CoV and MERS-CoV M ^pro^s in complex with PF-00835231 

**Figure 6:**
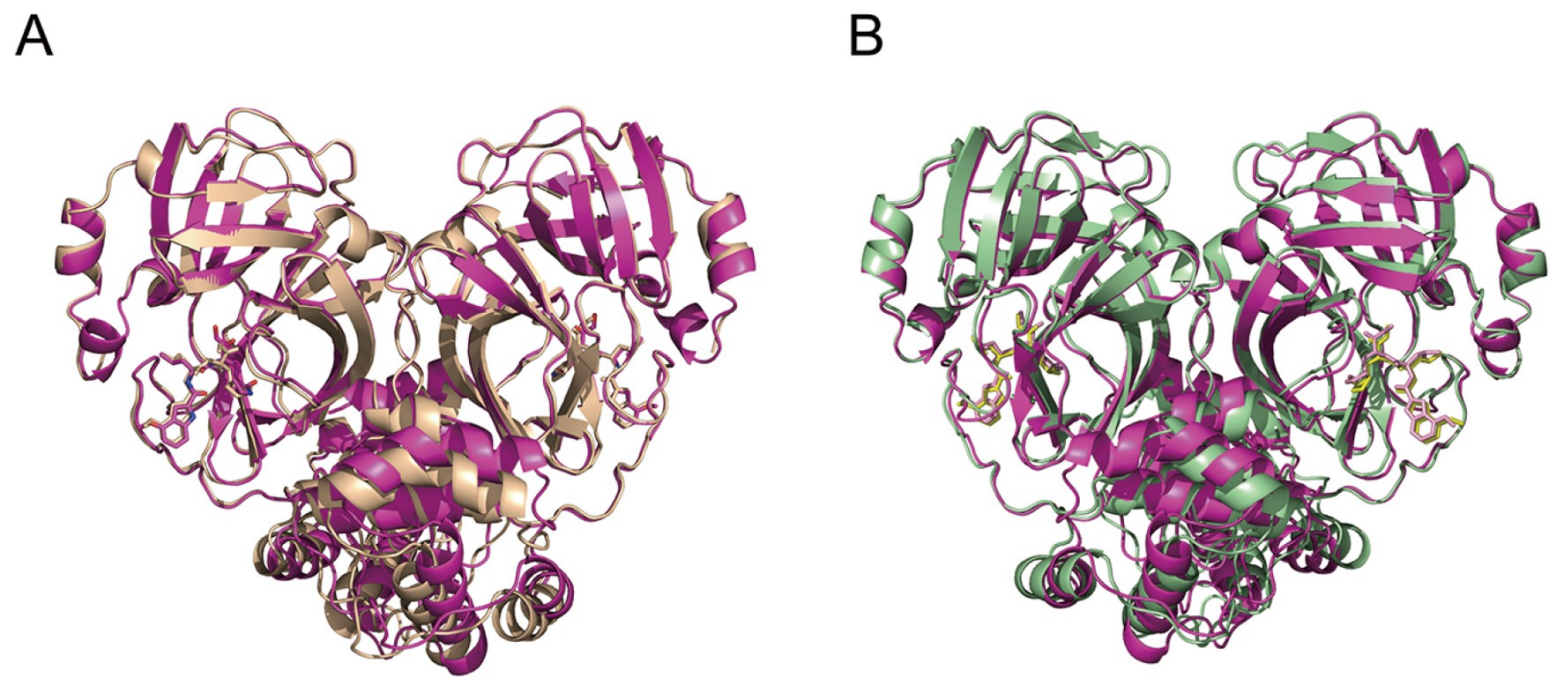
Figure 6 Structural comparison between different coronavirus M ^pro^s in complex with PF-00835231 (A) Structural comparison between SARS-CoV-2 Mpro-PF-00835231 (light magenta) and SARS-CoV M pro-PF-00835231 (wheat). (B) Structural comparison between SARS-CoV-2 M pro-PF-00835231 and MERS-CoV Mpro-PF-00835231 (pale green).

**Figure 7:**
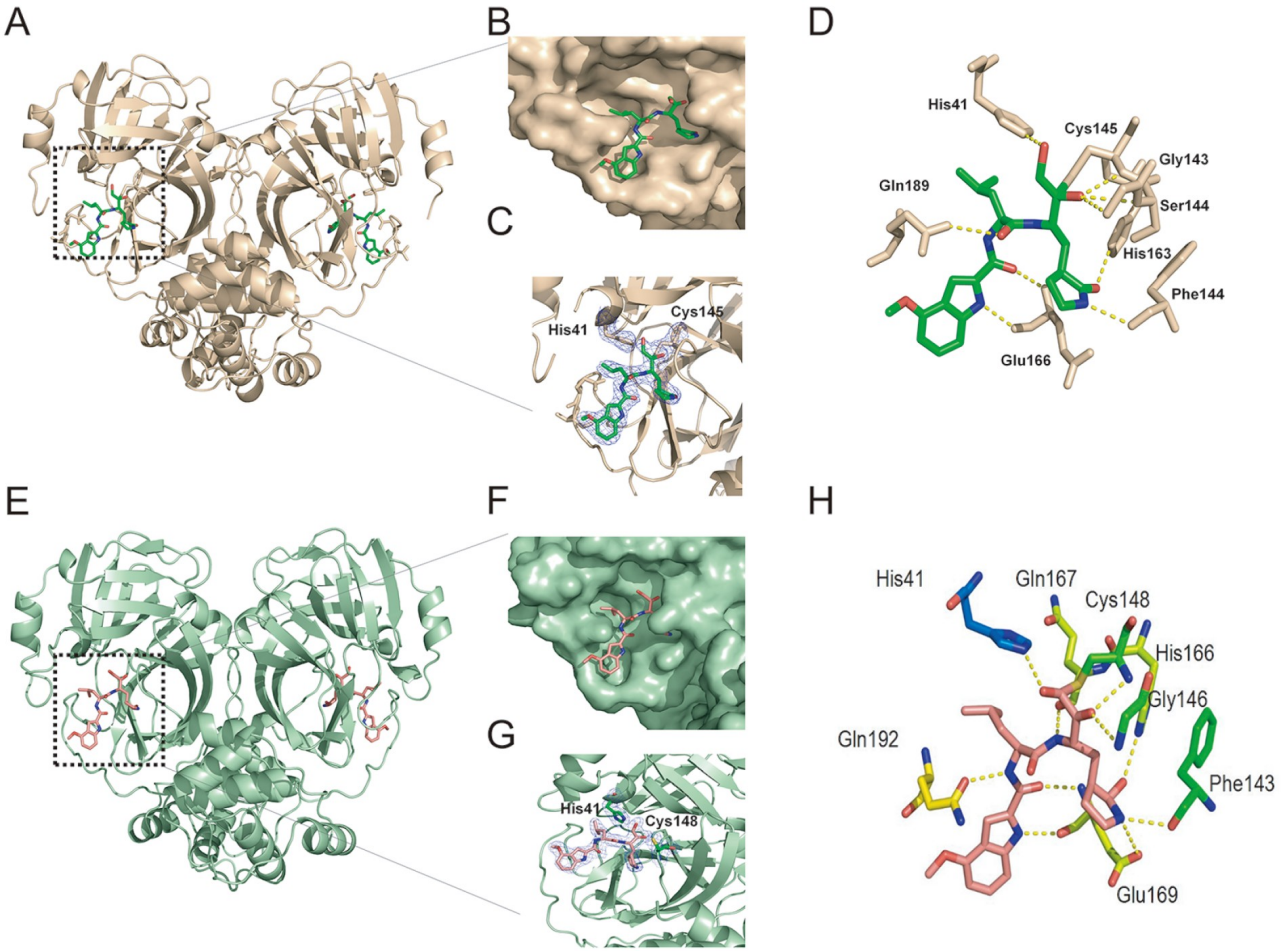
Figure 7 Crystal structures of SARS-CoV and MERS-CoV M ^pro^s in complex with PF-00835231 (A–D) SARS-CoV Mpro-PF-00835231 complex (wheat). (A) Overall structure of the SARS-CoV Mpro-PF-00835231 complex. Mpro is shown as a cartoon, and PF-00835231 is shown as a stick model. (B) PF-00835231 in subsites of the active site of SARS-CoV M pro. (C) 2Fo-Fc electron density map contoured at 1.0σ. (D) Interaction details (within 3.5 Å) between SARS-CoV Mpro and PF-00835231. Hydrogen bond interactions are depicted as dashed lines. (E–H) MERS-CoV Mpro-PF-00835231 complex (pale green). (E) Overall structure of the MERS-CoV Mpro-PF-00835231 complex. Mpro is shown as a cartoon. (F) PF-00835231 in subsites of the active site of MERS-CoV Mpro. (G) 2Fo-Fc electron density map contoured at 1.0σ. (H) Interaction details (within 3.5 Å) between MERS-CoV Mpro and PF-00835231. Hydrogen bond interactions are depicted as dashed lines.

We also resolved the crystal structures of PF-00835231 in complex with SARS-CoV and MERS-CoV M ^pro^s at resolutions of 2.61 Å and 2.30 Å, respectively ( Table 1). We then compared the structure of the SARS-CoV-2 M ^pro^-PF-00835231 complex with the structures of PF-00835231 in complex with SARS-CoV ( Figure 6A) and MERS-CoV ( Figure 6B) M ^pro^s. In terms of overall structure ( Figure 6), the M ^pro^s of SARS-CoV-2, SARS-CoV and MERS-CoV exhibit highly similar conformations when bound with PF-00835231. The RMSDs for the equivalent C-α positions range from 0.620 Å to 1.192 Å, respectively. We compared the crystal structures of SARS-CoV M ^pros^ ( Figure 7A–D) and MERS-CoV M ^pros^ ( Figure 7E–H) in complex with PF-00835231, including overall structure ( Figure 7A,E), the active site of M ^pro^ ( Figure 7B,F), 2Fo-Fc electron density map ( Figure 7C,G). As expected, PF-00835231 forms a C-S covalent bond with the sulfur atom of Cys145/Cys148, similar to that in the SARS-CoV-2 M ^pro^-PF-00835231 complex. We then analyzed the interactions (within 3.5 Å) between the SARS-CoV M ^pro^-PF-00835231 complex and the MERS-CoV M ^pro^-PF-00835231 complex ( Figure 7D,H). By comparison ( Figure 2G and 7D), we found that the ligand-enzyme binding modes are highly similar but with some differences. PF-00835231 directly forms hydrogen bonds with Gln89, Glu166, Phe140, His41, Ser144, Gly143, and His163 in SARS-CoV M ^pro^. However, residue His164 in SARS-CoV-2 M ^pro^ also forms hydrogen bonds with the methoxyketone group of PF-00835231. MERS-CoV M ^pro^ binds to PF-00835231 in a highly similar way to that of SARS-CoV-2 M ^pro^ ( Figure 2G and 7H). These observations provide a structural basis for how PF-00835231 binds with M ^pro^s from different coronaviruses and support that PF-00835231 can be used as a potent inhibitor with broad-spectrum potential to combat diseases caused by a variety of coronaviruses.

## Discussion

Over the past few years, the COVID-19 pandemic has continued to affect human health. Like other RNA viruses, SARS-CoV-2 is constantly changing through mutations, and each virus with a unique sequence is considered a new variant [29]. The continuous emergence of SARS-CoV-2 variants has seriously endangered public health. Continued research on these inhibitors is conducive to a rapid understanding of their current and future antiviral effects. Among several structural and non-structural SARS-CoV-2 proteins, M ^pro^ has been designated a potential therapeutic target for drug development [ 14, 16]. Inhibiting M ^pro^ prevents virus replication and is a potential anti-coronavirus strategy. The development of anti-coronavirus M ^pro^ inhibitors has attracted increased interest. A recombinant compound, the hydroxymethyl ketone covalent inhibitor PF-00835231, has entered clinical trials [17]. This phosphate prodrug, PF-07304814, is converted to its active form, PF-00835231, and irreversibly attaches to the active Cys. Here, we evaluated the interactions between the small hydroxymethylketone-based PF-00835231 molecule and seven different M ^pro^ mutants. 

Interestingly, in our study, the carbon on the methoxyl group of the indole group of PF-00835231 was found to interact with Thr190 in SARS-CoV-2 M ^pro^ but with Gln189 in M ^pro^ Y54C. However, we did not find that it interacts with any residues in other SARS-CoV-2 M ^pro^ mutants or in SARS-CoV M ^pro^ and MERS-CoV M ^pro^s, possibly because of their dynamic locations. Although the electron density is not high enough, the specific effect needs to be further verified, but it is certain that there is a certain interaction. This finding suggests some new possibilities for the mechanism of SARS-CoV-2 M ^pro^ inhibition by PF-00835231. 

In this study, we solved the crystal structure of PF-00835231 and SARS-CoV-2 M ^pro^ complexes and resolved the crystal structure of PF-00835231 with SARS-CoV and MERS-CoV M ^pro^s complexes. These structures suggest that PF-00835231 has similar binding patterns for different M ^pro^s but with subtle differences. These data update previous reports on the discovery of PF-00835231 and contribute to a comprehensive understanding of the inhibition mechanism of PF-00835231. Our study also determined the crystal structures of PF-00835231 in complex with several SARS-CoV-2 M ^pro^ mutants. Our data showed that the binding mode of PF-00835231 is not significantly affected by these mutants. The binding patterns of PF-00835231 with SARS-CoV-2 M ^pro^ mutants, SARS-CoV M ^pro^ and MERS-CoV M ^pro^s and SARS-CoV-2 variants are similar. 

Because M ^pro^ is highly conserved in different human coronaviruses, potent inhibitors such as PF-00835231 can also be used as broad-spectrum candidates for a variety of coronavirus infections. Therefore, this study provides a theoretical basis for the treatment of SARS-CoV-2 M ^pro^ mutants, SARS-CoV M ^pro^ and MERS-CoV M ^pro^s and SARS-CoV-2 variants and further drug development. 

## Supporting information

2-Supplementary_Materials

## Supplementary Data

Supplementary data is available at *Acta Biochimica et Biophysica Sinica* online.
