# Supplementary material for: Structural basis for the inhibition of coronaviral main proteases by PF-00835231: Crystal structures of M pro and M pro mutants bound to PF-00835231
Source: Acta Biochim Biophys Sin (Shanghai). 2024 Jul 29;56(12):1813–22. doi: 10.3724/abbs.2024122 (PMC11693862; doi:10.3724/abbs.2024122)
Supplement: 2-Supplementary_Materials [file 2-Supplementary_Materials.pdf]

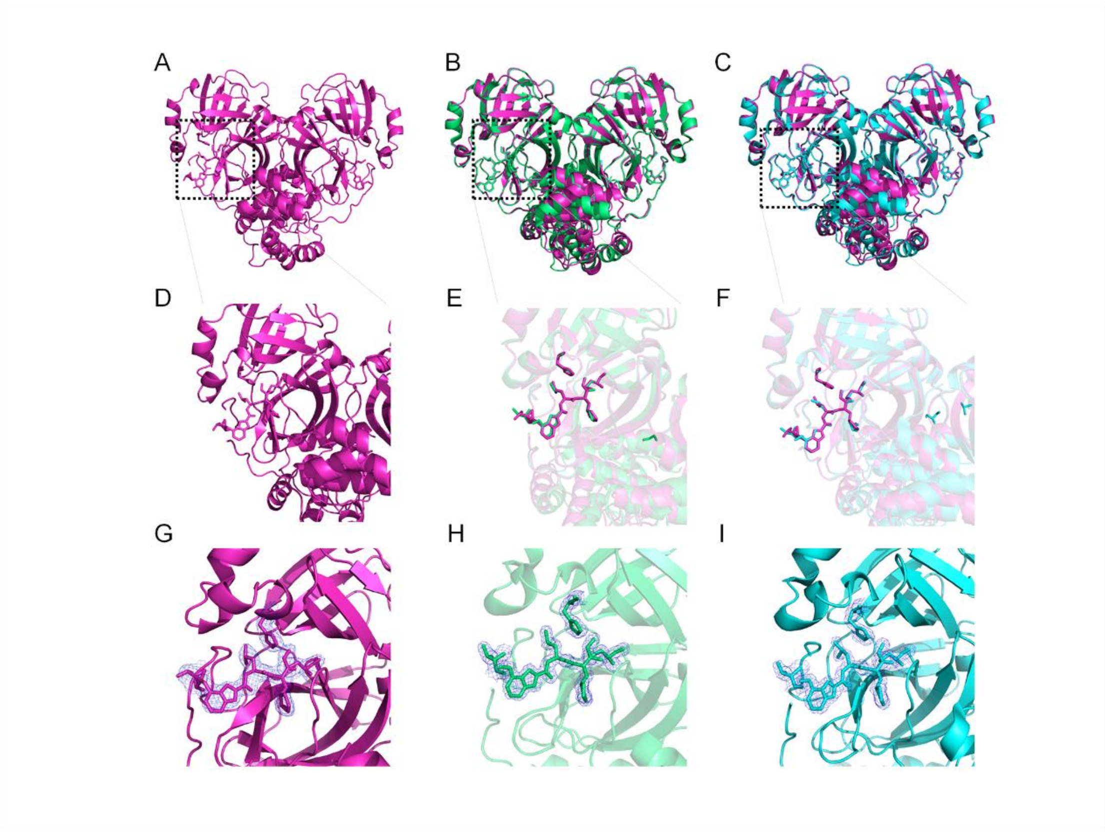

Supplementary Figure S1. Structural comparison between SARS-CoV-2 M^{pro}-PF-00835231 and available structures (A) Structure of SARS-CoV-2 M^{pro}-PF-00835231. (B) Structural comparison between SARS-CoV-2 M^{pro}-PF-00835231 and SARS-CoV-2 M^{pro}-PF-00835231 (PDB ID 6XHM, Lime green). (C) Structural comparison between SARS-CoV-2 M^{pro}-PF-00835231 and SARS-CoV-2 M^{pro}-PF-00835231 (PDB ID 8DSU, cyan). SARS-CoV-2 M^{pro}-PF-00835231 is shown as a cartoon with light magenta (this research), lime green (PDB ID 6XHM) and cyan (PDB ID 8DSU). (D–F) The zoomed-in view of the substrate binding pocket of the main proteases. PF-00835231 and its catalytic dyad residues are shown as sticks. (G) The 2*Fo-Fc* electron density map (contoured at 1.0σ) of the inhibitor and catalytic dyad residues His41, Cys145 and Thr190 in the SARS-CoV-2 M^{pro}-PF-00835231 complex (this study) is shown as blue mesh. (H) The 2*Fo-Fc* electron density map (contoured at 1.0σ) of the inhibitor and catalytic dyad residues His41, Cys145 and Thr190 in the SARS-CoV-2 M^{pro}-PF-00835231 complex (PDB ID 6XHM) is shown as a blue mesh. (I) The 2*Fo-Fc* electron density map (contoured at 1.0σ) of the inhibitor and catalytic dyad residues His41, Cys145 and Thr190 in the SARS-CoV-2 M^{pro}-PF-00835231 complex (PDB ID 8DSU) is shown as a blue mesh.

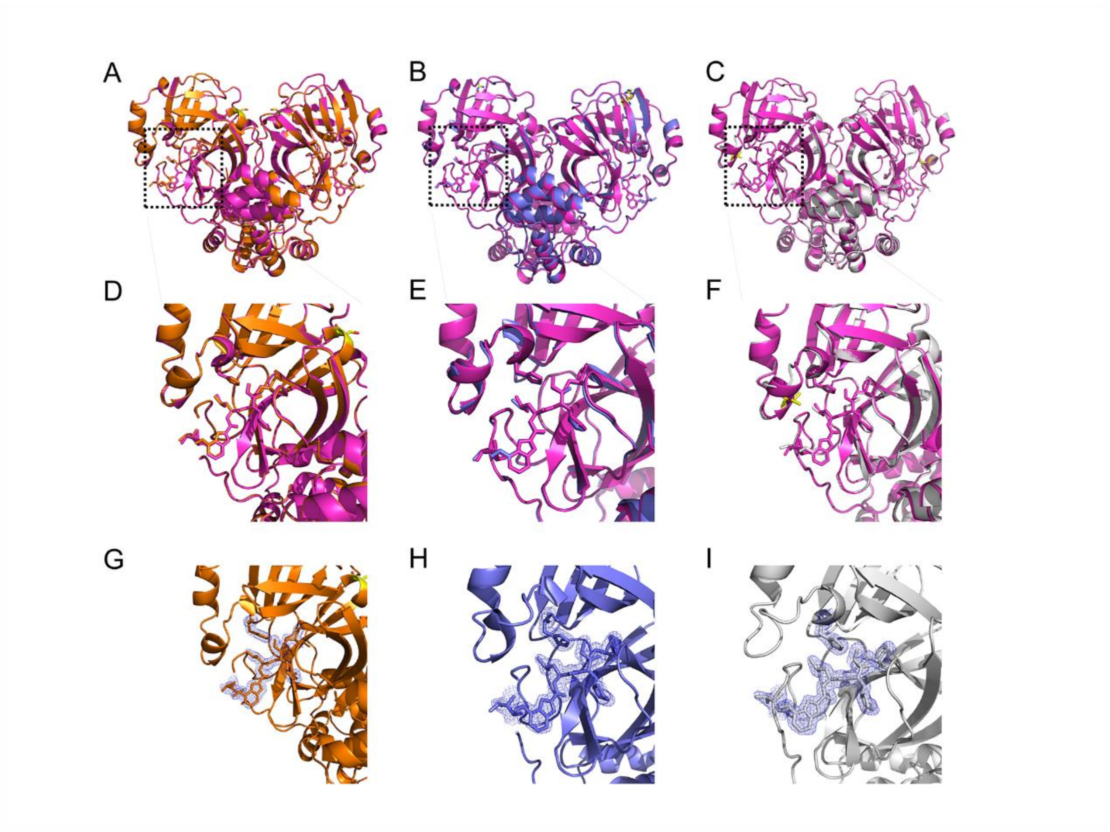

Supplementary Figure S2. Structural comparison between SARS-CoV-2 M^{pro}-PF-00835231 and the M^{pro} mutants PF-00835231 (G15S, K90R and M49I). S15, R90 and I49 are shown as yellow sticks (A) Structural comparison between SARS-CoV-2 M^{pro}-PF-00835231 and G15S-PF-00835231. (B) Structural comparison between SARS-CoV-2 M^{pro}-PF-00835231 and M^{pro} K90R-PF-00835231. (C) Structural comparison between SARS-CoV-2 M^{pro}-PF-00835231 and M^{pro} M49I-PF-00835231. (D–F) The zoomed-in view of the substrate binding pocket of main proteases. PF-00835231 and catalytic dyad residues are shown as sticks. (G–H) 2*Fo-Fc* electron density maps of PF-00835231 bound to different SARS-CoV-2 M^{pro} mutants. 2*Fo-Fc* electron density maps (blue) were constructed at 1σ. (G) The 2*Fo-Fc* electron density map of PF-00835231 bound to the G15S mutant. (H) The 2*Fo-Fc* electron density map of PF-00835231 bound to the K90R mutant. (I) The 2*Fo-Fc* electron density map of PF-00835231 bound to the M49I mutant.

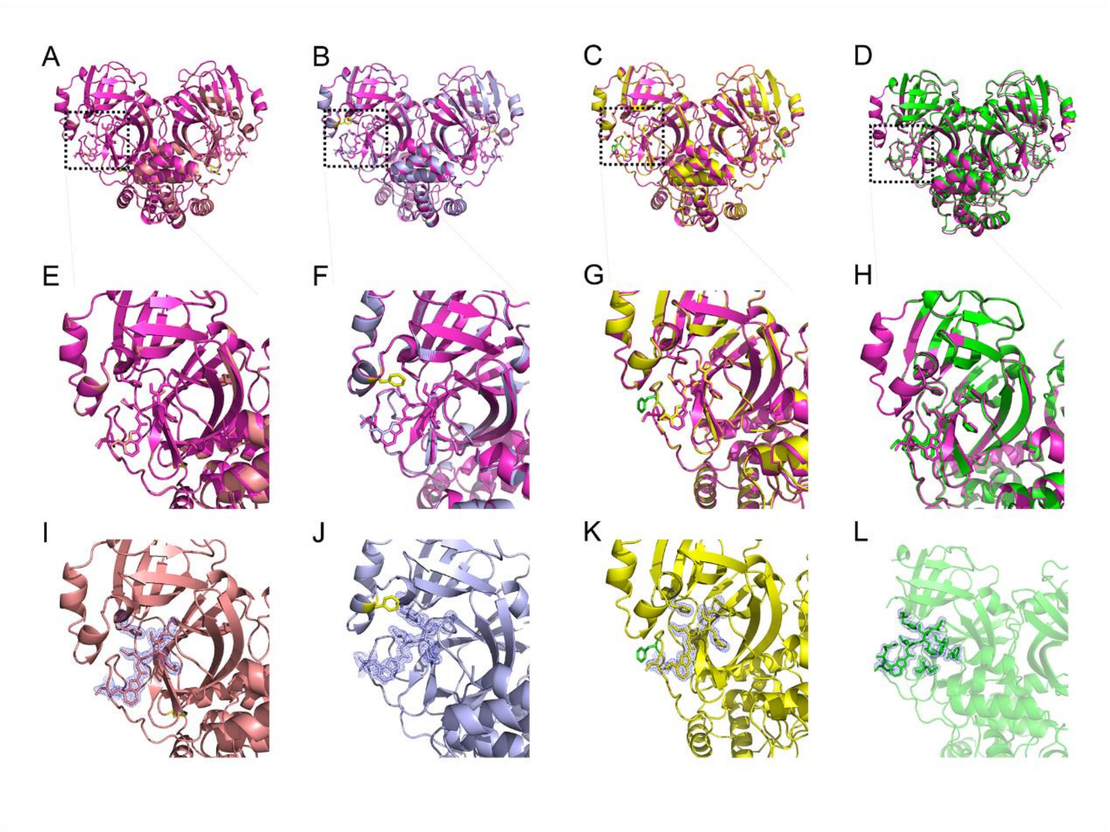

Supplementary Figure S3. Structural comparison between SARS-CoV-2 M^{pro}-PF-00835231 and the M^{pro} mutant PF-00835231 (P132H, S46F, V186F and Y54C). H132, F46 and C54 are shown as yellow sticks, while F186 is shown as green sticks (A) Structural comparison between SARS-CoV-2 M^{pro}-PF-00835231 and P132H-PF-00835231. (B) Structural comparison between SARS-CoV-2 M^{pro}-PF-00835231 and M^{pro} S46F-PF-00835231. (C) Structural comparison between SARS-CoV-2 M^{pro}-PF-00835231 and M^{pro} V186F-PF-00835231. (D) Structural comparison between SARS-CoV-2 M^{pro}-PF-00835231 and M^{pro} Y54C-PF-00835231. (E–H) The zoomed-in view of the substrate binding pocket of main proteases. PF-00835231 and catalytic dyad residues are shown as sticks. (I–L) 2*Fo-Fc* electron density maps of PF-00835231 bound to different SARS-CoV-2 M^{pro} mutants. 2*Fo-Fc* electron density maps (blue) were contoured at 1σ. (I) The 2*Fo-Fc* electron density map of PF-00835231 bound to the P132H mutant. (J) The 2*Fo-Fc* electron density map of PF-00835231 bound to the S46F mutant. (K) The 2*Fo-Fc* electron density map of PF-00835231 bound to the V186F mutant. (L) The 2*Fo-Fc* electron density map of PF-00835231 bound to the Y54C mutant.
